# Treatment Satisfaction and Its Influencing Factors in Parkinson's Disease: A Web-Based Survey of Patients and Physicians in Clinical Practice in Japan

**DOI:** 10.1155/2022/2732021

**Published:** 2022-02-23

**Authors:** Masahiro Nomoto, Ayako Hayashi, Hiroyuki Ida, Masaki Arai

**Affiliations:** ^1^The Social Welfare Organization Imabari Center for Health and Welfare, 7-1-6 Kitamura, Imabari, Ehime 799-1502, Japan; ^2^Japan Medical Office, Takeda Pharmaceutical Company Limited, 1-1 Nihonbashi-Honcho 2-chome Chuo-ku, Tokyo 103-8688, Japan

## Abstract

**Objective:**

This study aimed to gain an understanding of patient and physician satisfaction with overall treatment and routine consultations for Parkinson's disease in clinical practice.

**Methods:**

This observational, cross-sectional, web-based survey was conducted in Japan from February to March 2019. Eligible patients with Parkinson's disease (*N* = 186) and physicians who treat patients with Parkinson's disease (*N* = 331) were asked to evaluate their satisfaction with treatment, consultation, symptom control, and use of a symptom diary.

**Results:**

Patients had a mean age of 62.7 years, 54.8% were male, and most (75.8%) had Hoehn and Yahr stage ≥3 symptoms. Physicians were mostly male (93.1%) and had treated 52 patients with Parkinson's disease in the last 6 months, and 34.1% were certified neurologists. There were significant gaps between patient and physician satisfaction with treatment and consultations. Patient and physician satisfaction with overall treatment was significantly lower for patients with Hoehn and Yahr stage ≥3 symptoms than stage 1-2 symptoms (patients: 53.9% vs. 71.1%; physicians: 43.2% vs. 69.7%, respectively). The proportion of patients who were satisfied with symptom control was lower than that of physicians (26.4% vs. 51.5%). Influencing factors for patient satisfaction with treatment were nonmotor symptoms (e.g., insomnia and depression). Satisfaction tended to be higher for patients and physicians when symptom diaries were used.

**Conclusion:**

Significant gaps in perceptions of treatment and consultation exist between patients and physicians in Parkinson's disease. Physicians should participate in shared decision making with their patients and consider strategies for management of nonmotor symptoms and nonpharmacological therapies and encourage the use of symptom diaries.

## 1. Introduction

The global burden of Parkinson's disease, which is one of the fastest-growing neurological diseases worldwide, increased more than two-fold from 1990 to 2016, in part because of aging populations and increased life expectancy [1]. In Japan, which has a rapidly aging population, the number of patients with Parkinson's disease increased from 75,000 in 1987 to 162,000 in 2017 [2]. At present, no drugs with neuroprotective effects have been developed for Parkinson's disease, and patients require long-term multidisciplinary care as both motor and nonmotor symptoms emerge and gradually worsen over time [3]. In addition, because symptomatology is heterogenous, management of patients with Parkinson's disease requires tailored therapies that focus on the most common symptoms experienced by individual patients [3, 4]. Communication between patients and their physicians is critically important for ensuring that patients receive the most appropriate information and have access to the most appropriate care throughout their disease journey [5–7].

Although patient-physician communication is a significant contributor to patient satisfaction and improvements in quality of life in Parkinson's disease [8, 9], very few studies have been conducted to assess patient satisfaction with treatment and the factors that contribute to patient satisfaction [7, 10]. Findings from a survey of patients in the United States showed that although patients were satisfied with the time spent with their practitioners and the information they received on Parkinson's disease, they were less satisfied with the information received on disease progression and nondrug treatments [10]. Furthermore, there is limited information on the alignment between patient and physician perspectives on treatment satisfaction and decision making in Parkinson's disease [6, 11, 12]. Two-way surveys of patients and physicians conducted in the United States and Europe suggested that patients place greater emphasis on nonmotor symptoms and modes of medication delivery and are less confident in their knowledge of support services than physicians [11–14]. However, there is little information on the alignment of patient-physician perspectives of treatment in Japan [6].

The objective of this study was to gain an understanding of patient and physician satisfaction with overall treatment and routine consultations for Parkinson's disease in clinical practice in Japan using a web-based survey. In addition, we aimed to assess patient and physician satisfaction with symptom control and identify the factors associated with patient and physician satisfaction by exploring patient and physician perceptions of communication during consultations at the time of diagnosis and during routine treatment.

## 2. Methods

### 2.1. Study Design

This was an observational, cross-sectional, web-based survey conducted in Japan from February to March 2019. Participants were recruited via market research agencies (Macromill Carenet, Inc.). A screening questionnaire was distributed via e-mail to 355,765 Macromill registrants and 19,550 Carenet registrants to identify eligible patients and physicians, respectively, at the time of distribution of the questionnaires. Patients who completed the screening questionnaire (February 15–22, 2019) and fulfilled the eligibility criteria for the survey were then invited by e-mail to participate in the main questionnaire (March 5–12, 2019). Physician participants completed the screening and main questionnaire on March 5–8, 2019.

The protocol was approved by the Research Institute of Healthcare Data Science research ethics committee (RI2018014) in Japan. All data in the survey were anonymized, and all participants provided consent for their responses to be used for medical research purposes. All patients and physicians were provided with the study details at the start of the questionnaire and gave electronic informed consent to participate. Participants could discontinue at any time during the questionnaire. The survey was developed and delivered in Japanese. The study was registered at the UMIN Clinical Trials Registry (https://www.umin.ac.jp/ctr/index.htm (UMIN000035769)).

### 2.2. Study Population

The inclusion criteria for patients were as follows: male and female, aged ≥20 years; patients with a diagnosis of Parkinson's disease or a family member and cohabitant who could act on a patient's behalf; reported they attended regular hospital visits for treatment of Parkinson's disease; and reported at least one symptom among the four major symptoms of Parkinson's disease (postural instability, tremor, rigidity, and bradykinesia). Patients (identified by the screening questionnaire) who did not report any of the four main major symptoms of Parkinson's disease, had no regular hospital visits for treatment of Parkinson's disease, had comorbidities or dementia, or who provided answers that were illogical or inconsistent were excluded. To eliminate subjective assumptions, responses by patients' family members were excluded if they were evaluated objectively; only responses that were derived from a direct interview were analyzed.

The inclusion criteria for physicians were as follows: neurologists who had examined 25 or more patients with Parkinson's disease in the last 6 months; neurosurgeons who had examined 5 or more patients with Parkinson's disease in the last 6 months; and general internal physicians or psychiatrists who had examined 10 or more patients with Parkinson's disease in the last 6 months. Physicians who were not responsible for patients' treatment strategies or who provided answers that were illogical or inconsistent were excluded.

### 2.3. Outcome Measures and Endpoints

#### 2.3.1. Questionnaires and Assessment

The screening questionnaire comprised 10 questions for patients and nine questions for physicians, and the main questionnaire comprised 33 questions for patients and 31 questions for physicians (Supplementary Tables S1-1 and S1-2). Family members who acted on behalf of patients were asked to directly enter responses from patients at the time the questionnaire was completed. The Hoehn and Yahr (H&Y) staging scale (self-reported) was used to classify the severity of Parkinson's disease symptoms and disability, where an H&Y of 1-2 referred to mild symptoms and an H&Y ≥ 3 referred to moderate-to-severe symptoms.

#### 2.3.2. Satisfaction with Factors Related to Treatment

There were 15 items related to satisfaction of the overall consultation, overall treatment, pharmacotherapy, exercise therapy/rehabilitation, medical support, and medical expenses subsidies. These included five items on the consultation: consultation hours, comprehensibility of explanation, treatment strategy/decision, communication, and overall consultation (comprehensive evaluation of four items); four items on pharmacotherapy: medication effectiveness (improvement/prevention of symptoms and time to be effective), side effects (severity and impact on daily life), convenience (ease of taking the medication and frequency of administration), and overall pharmacotherapy (comprehensive evaluation of three items); three items on exercise therapy/rehabilitation: guidance (comprehensibility and frequency of the guidance), effectiveness (improvement/prevention of symptoms), and overall exercise therapy/rehabilitation (comprehensive evaluation of two items); one item on medical support from healthcare professionals, which included support provided by nurses, pharmacists, and physical therapists (this item was applied to patients only); and one item on medical expenses, which included the expense of consultation, medication, rehabilitation, and other treatment (this item was applied to patients only). Overall treatment was assessed by combining responses to the pharmacotherapy and exercise therapy/rehabilitation items. Participants rated their satisfaction on a 6-point scale where 1 = extremely dissatisfied, 2 = dissatisfied, 3 = slightly dissatisfied, 4 = slightly satisfied, 5 = satisfied, and 6 = extremely satisfied. A score of 4–6 was classified as “satisfied” and 1–3 was “dissatisfied.”

#### 2.3.3. Consultation Content

Patients and physicians were asked to provide additional information on their perceptions of the consultation at the time of diagnosis of Parkinson's disease and during routine treatment. Patients and physicians were asked to select, from a list of topics, what had been discussed at the time of diagnosis and during routine treatment. For each topic, patients were asked if they felt satisfied (understood) or dissatisfied (wished to receive more information). There were 11 topics at the time of diagnosis (causes and mechanism of disease, characteristic symptoms, progression of the disease, how to deal with worsening symptoms, effects and side effects of medications, exercise therapy/rehabilitation, surgical treatment, what lifestyle factors patients should be aware of, social support (care service or others), and patient associations) and 13 topics for the routine consultations (change in symptoms, appearance of new symptoms, impact of symptoms on daily life, concerns and questions about symptoms, concerns and questions about overall treatment, setting treatment goals, effect of medication, side effects of medication, forgetting to take the medication, desire to change the medication, how and how often to use rehabilitation at hospital, frequency and procedure of exercise at home, and others).

#### 2.3.4. Satisfaction with Symptom Control

There were 26 items related to satisfaction with symptom control. These included eight items on motor symptoms: tremor, rigidity, bradykinesia, postural instability, freezing of gait, dyskinesia, and difficulty with handwriting and speech; two items on sleep: insomnia and daytime sleepiness; two items on urination and defecation: urgent or nocturnal urination and constipation; one item on cognitive impairment (forgetfulness and cannot concentrate on anything); two items on mood or motivation: apathy (no interest and no motivation) and depression; two items on eating and gastrointestinal symptoms: difficulty swallowing and stomach upset and/or nausea; two items on pain: dystonia (muscle stiffness or pain during an off period) and back and joint pain; one item on hallucination and visual hallucination; one item on impulse control disorders: have an urge to gamble, shop, or play games and have jealousy or delusion; one item on wearing off (control of medication effectiveness): duration of effectiveness of medication may be shorter than when the medication was effective or stable, and symptoms of Parkinson's disease may appear before the next administration; and four items on other symptoms: dizziness, olfactory disturbances, excessive sweating, and fatigue (feeling tired regularly). For symptom control, participants rated their satisfaction with the control of their symptoms on a 4-point scale where 1 = extremely dissatisfied, 2 = dissatisfied, 3 = satisfied, and 4 = extremely satisfied. A score of 3 and 4 was classified as “satisfied” and 1 and 2 was “dissatisfied.”

#### 2.3.5. Use of a Symptom Diary

There was one item on the use of a symptom diary (paper or smartphone app or other). Patients were asked about the frequency of use (continuously, sometimes, used in the past (not current), never used although I knew about it, or I do not know about symptom diaries). Physicians were asked about how they instructed patients to use the diary (instructed to use the diary on a daily basis, use the diary temporarily for adjustment of medication, rarely use, or never use).

The endpoints included in this analysis were as follows: the proportion of patients and physicians who were “satisfied” with the consultation and with overall treatment, pharmacotherapy, exercise therapy/rehabilitation, general treatment, and support from healthcare professionals; patient and physician satisfaction scores for the consultation, pharmacotherapy, exercise/rehabilitation therapy, overall treatment, medical support, and medical expenses; the proportion of patients and physicians who were satisfied (extremely satisfied or satisfied) with symptom control (motor or nonmotor); and the proportion of patients who did and did not use a symptom diary who were satisfied (extremely satisfied or satisfied) with each symptom.

### 2.4. Statistical Analysis

The analysis population included all patients and physicians who met the inclusion criteria.

Comparisons between the proportions of patients and physicians who were “satisfied” and between patients with mild symptoms (H&Y 1-2) and moderate-to-severe symptoms (H&Y ≥ 3) were conducted using a chi-square test with 95% confidence intervals. A given alpha value of 0.05/4 = 0.0125 was used based on Bonferroni correction for the analyses of satisfaction. Evaluation of the correlation between satisfaction scores and various factors such as age, sex, and with/without symptoms was conducted using a bivariate analysis. The normal distribution was tested using an F-test, and the mean differences (±standard deviation) between groups were assessed using Student's *t*-test (homoscedasticity of variance) or a Welch *t*-test (heteroscedasticity of variance). Satisfaction with symptom control and type and use of a symptom diary was assessed using scatter plots. All analyses were conducted using the Statistical Package for the Social Sciences (IBM, version 26, Armonk, NY, USA) and *R* version 4.0.2 (the *R* Foundation for Statistical Computing Platform, Vienna, Austria).

## 3. Results

### 3.1. Study Population

Of the 355,765 available registrants, 1393 were identified from the screening questionnaire as patients with Parkinson's disease (Supplementary Figure 1). Of the 626 who were deemed eligible and invited to participate in the main questionnaire, 411 responded to the main questionnaire and 186 met the inclusion criteria for the analyses. The main reasons for exclusion from the analyses were cohabiting family members who did not directly interview the patient with Parkinson's disease and no major symptoms. Of the included patients, 54.8% were male with a mean age of 62.7 years. The mean age at diagnosis of Parkinson's disease was 56.2 years, the mean duration of disease since diagnosis was 6.5 years, and 75.8% of patients had moderate-to-severe symptoms (self-reported H&Y ≥ 3) (Table 1). Most patients (76.3%) were being treated in a neurology department, 36.0% visited their medical institution once a month, and 34.4% visited once every 2 or 3 months. Wearing off was experienced by 60.8% of patients.

Of the 19,550 available Carenet registrants, 1597 registrants visited the site, 348 physicians answered the main questionnaire, and 331 met the inclusion criteria (Supplementary Figure 1). Almost all physicians were male with a mean of 19.4 years' experience (Table 2). On average, physicians had treated 52.1 patients with Parkinson's disease in the last 6 months, approximately one-third (34.1%) were neurologists certified by the Japanese Society of Neurology, and most were working in a general internal medicine or neurology department.

### 3.2. Overall Satisfaction with Consultation and Treatment

**Figure 1:**
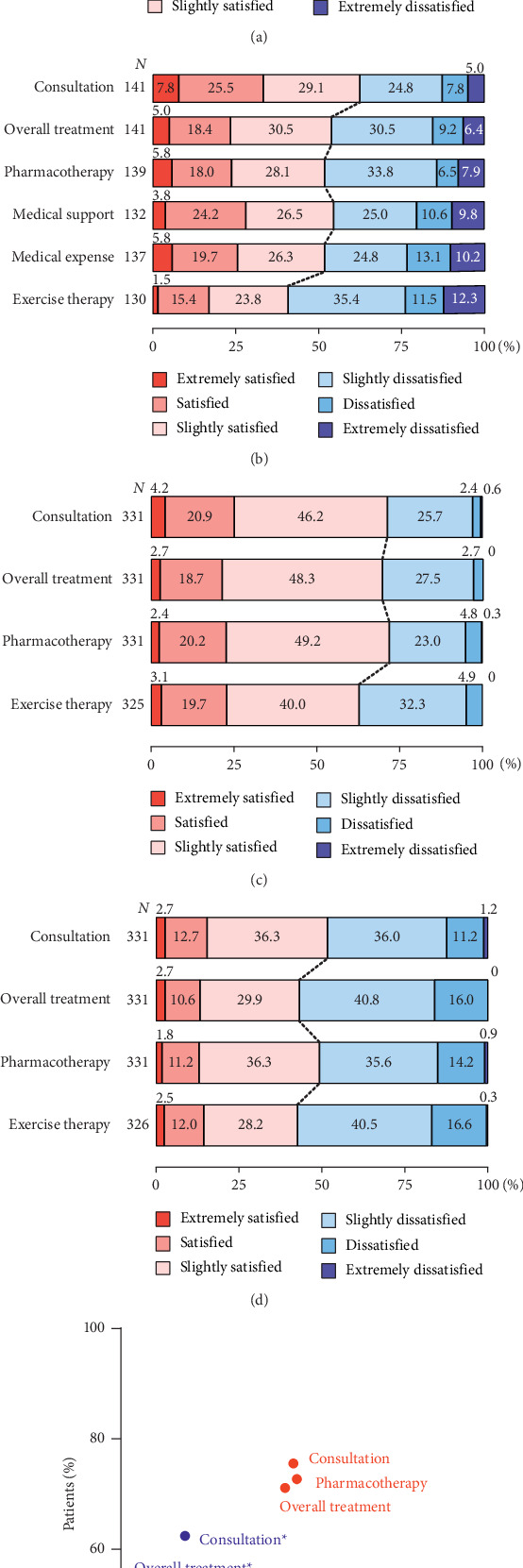
Overall patient and physician satisfaction with consultation and treatments for Parkinson's disease by patient disease severity (H&Y stage). (a) Patients (self-reported H&Y 1-2). (b) Patients (self-reported H&Y ≥ 3). (c) Physicians (H&Y 1-2). (d) Physicians (H&Y ≥ 3). (e) Patients and physicians by H&Y stage. ⁣^*∗*^*p* < 0.05, significant difference between patients and physicians. H&Y: Hoehn and Yahr.

Satisfaction with all aspects of consultation and treatment tended to be lower for patients with moderate-to-severe symptoms (self-reported H&Y ≥ 3) and physicians of patients with moderate-to-severe symptoms (H&Y ≥ 3) compared with mild symptoms (H&Y 1-2) (Figure 1). Physicians of patients with moderate-to-severe symptoms (H&Y ≥ 3) (Figure 1(d)) reported significantly lower satisfaction (slightly satisfied, satisfied, and extremely satisfied) than patients (self-reported H&Y ≥ 3) (Figure 1(b)) with the consultation (51.7% vs. 62.4%; *p* < 0.01) and overall treatment (43.2% vs. 53.9%; *p* < 0.01). For patients, there were no notable differences in satisfaction with the overall consultation between patients with mild (self-reported H&Y 1-2) and moderate-to-severe (self-reported H&Y ≥ 3) symptoms (75.6% vs. 62.4%, respectively). Of all the factors assessed, patients and physicians were least satisfied with exercise therapy/rehabilitation, irrespective of disease severity. There were no notable differences in the proportions of physicians and patients who were satisfied in the mild (H&Y 1-2, self-reported for patients) symptom groups, but there was a tendency toward lower rates of satisfaction among physicians compared with patients in the moderate to-severe (H&Y ≥ 3, self-reported for patients) symptom group (Figure 1(e)).

### 3.3. Factors Associated with Patient and Physician Satisfaction

For patients, the factors found by bivariate analysis to be significantly associated with satisfaction scores across all categories were insomnia, depression, and fatigue, and all categories except exercise therapy/rehabilitation were stomach upset and olfactory disturbance (Supplementary Table S2). Additional factors significantly associated with lower satisfaction with the consultation were difficulty with speech, cognitive impairment, and apathy. Lower satisfaction scores for exercise therapy/rehabilitation were significantly associated with patients aged <60 years and symptoms of insomnia, depression, impulse control disorders, and fatigue. Among physicians, the factors that were significantly associated with higher satisfaction scores were neurology specialist, seeing more patients (≥50 patients in the last 6 months), and membership of a neurological society, irrespective of the patient's disease severity (Supplementary Table S3).

### 3.4. Patient and Physician Perceptions during Consultations at the Time of Diagnosis and during Routine Treatment

**Figure 2:**
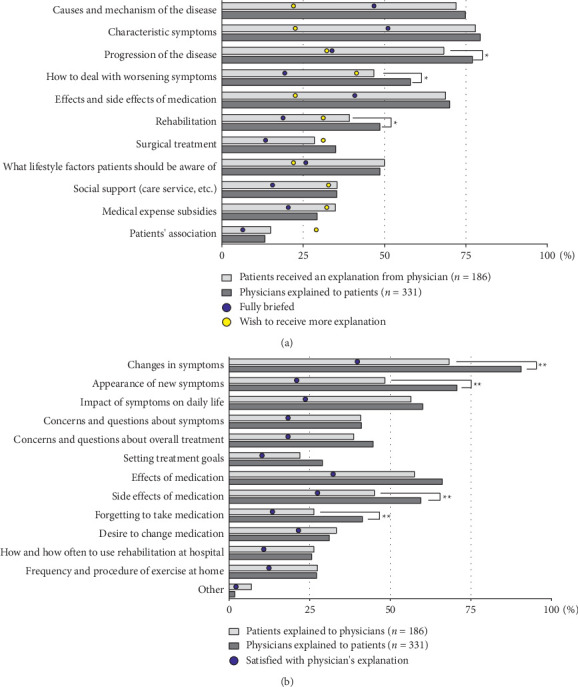
Consultation discussion topics. (a) What was discussed during the consultation at PD diagnosis (patient- and physician-reported)? (b) What was discussed during routine consultations for treatment of PD (patient- and physician-reported)? ⁣^*∗*^*p* < 0.05, ⁣^*∗∗*^<0.01, significant difference between patients and physicians. PD: Parkinson's disease.

There was a high level of alignment between the topics that patients received information on and for which physicians gave information at the time of diagnosis (Figure 2(a)). The top three topics that were consistently reported by >50% of patients and physicians were causes and mechanism of disease (72.0%, 74.9%, respectively), symptom characteristics (78.0%, 79.5%), and progression of disease (68.3%, 77.0%) (Figure 2(a)). Gaps between patients and physicians were observed for progression of disease (68.3%, 77.0%), how to deal with worsening symptoms (46.8%, 58.0%), and rehabilitation (39.2%, 48.6%). For progression of the disease, 33.9% of patients were fully satisfied with their physicians' explanations, but 41.4% of patients wished to receive more information on how to deal with worsening symptoms (Figure 2(a)).

There were significant gaps between patients and physicians in the perceptions of what was discussed during routine consultations (Figure 2(b)). Physicians reported discussing (hearing/explaining) patients' changes in symptoms (90.6%), emergence of new symptoms (70.7%), and side effects of medications (59.5%) and forgetting to take medication (41.4%), but the proportion of patients who reported discussing changes in symptoms (68.3%) and emergence of new symptoms (48.4%) with their physicians was low (Figure 2(b)). In addition, there were gaps between patients and physicians for the side effects of medications (45.2%, 59.5%, respectively) and forgetting to take medications (26.3%, 41.4%, respectively). Patients were not satisfied with the information they received from physicians on most topics (Figure 2(b)). In particular, satisfaction with explanations about setting treatment goals (10.2%), forgetting to take medication (13.4%), and rehabilitation at the hospital (10.5%) or at home (12.4%) was low.

### 3.5. Satisfaction with Symptom Control

**Figure 3:**
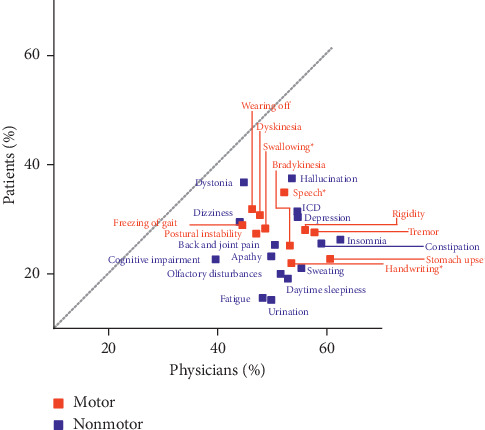
Satisfaction with symptom control by motor or nonmotor; “satisfied” includes “extremely satisfied” and “satisfied.” ⁣^*∗*^Difficulties with these functions. ICD: impulse control disorders.

Satisfaction with the control of each symptom was lower among patients than physicians (Supplementary Figure 2). The mean proportion of patients who were satisfied with their symptom control was 26.4%, which was approximately half that of physicians (51.5%). Patients were least satisfied with their nonmotor symptom control, specifically urination, fatigue, daytime sleepiness, and olfactory disturbances (≤ 20% satisfied) (Figure 3). The gaps between patient and physician ratings of satisfaction with symptom control were greatest mainly for the nonmotor symptoms of insomnia, urination, sweating, daytime sleepiness, constipation, fatigue, and olfactory disturbances (Figure 3).

**Figure 4:**
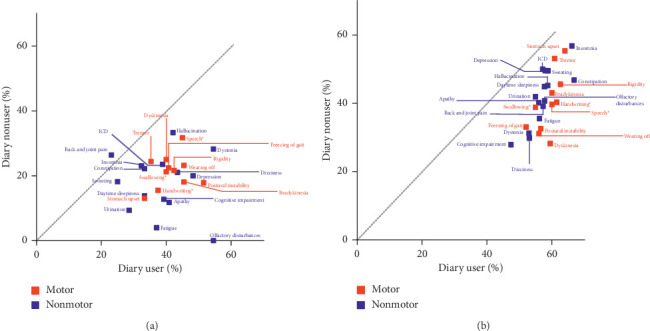
Satisfaction with symptom control with or without a symptom diary. (a) Patients. (b) Physicians. ⁣^*∗*^Difficulties with these functions. ICD: impulse control disorders.

The proportion of patients who were satisfied with symptom control tended to be higher among diary users than diary nonusers (Figure 4). Patient satisfaction with nonmotor symptoms (particularly olfactory disturbances, fatigue, apathy, depression, and cognitive impairment) and motor symptoms (particularly postural instability, bradykinesia, and dystonia) was higher among diary users than nonusers. Physician satisfaction with symptom control was slightly higher for diary users than nonusers. In particular, physician satisfaction with motor symptoms (especially dyskinesia) was higher among diary users than nonusers.

## 4. Discussion

The findings from this web-based survey have provided clinically relevant insights into patient and physician satisfaction with treatment of Parkinson's disease and highlight the different perspectives of patients and physicians during treatment. Patient and physician satisfaction with all aspects of consultation and treatment tended to be lower for patients with moderate-to-severe symptoms than mild symptoms. Physicians were less satisfied than patients with consultations and overall treatment for patients with moderate-to-severe symptoms, and patients were less satisfied than physicians with motor and nonmotor symptom control. However, both patients and physicians were more satisfied with motor and nonmotor symptom control for those who used a symptom diary compared with those who did not. Overall, these data have important implications for ways in which physicians can improve their communication with, and management of, patients with Parkinson's disease.

In general, there was good alignment between patients and physicians on information communicated at diagnosis on the causes and mechanism of the disease, characteristic symptoms, and effects and side effects of medication. However, exercise therapy/rehabilitation, surgical treatment, social support, medical expense subsidies, and patients' association were less frequently discussed despite almost one-third of patients wishing they had received more information on these topics. Importantly, the greatest areas of disconnect between patients and physicians were progression of the disease, how to deal with worsening symptoms, exercise therapy/rehabilitation at diagnosis, changes in symptoms, emergence of new symptoms, side effects of medication, and forgetting to take medication at routine clinical visits. These findings are consistent with a survey of patients in the United States, which showed that patients are less likely to be satisfied with the information they receive on prognosis and nonpharmacotherapies for Parkinson's disease [10], a matched patient-physician study in the United States, which showed a similar disconnect between patients and physicians on patients' understanding of their disease and the availability of nonmedical support [12], and studies conducted in Europe [7] and Japan [6], which showed that patients are looking for a personalized approach and are more satisfied when they are involved in treatment decisions. Despite these similarities, not all of the physicians in our study were specialized for treating patients with PD. Therefore, satisfaction between patients and physicians may have been different if the study were conducted with physicians from a tertiary center such as a PD-specific medical center. One of the reasons for a patient perceived lack of information from physicians is that physicians may not provide information on treatments or other interventions (e.g., surgery) that are not effective or applicable for a particular patient. However, it is more likely that detailed explanation of the complexities of treating Parkinson's disease is difficult in the time available during neurological appointments, and physicians may not have all the information that patients require during the consultation. As recommended by the National Institute for Health and Care Excellence guidelines [5], assignment of an additional healthcare professional (e.g., nurse specialist, social worker, and therapist) who can provide guidance to patients on the full range of services available will help ensure patients have access to the information and support they need.

A key finding from this study was that patients and physicians were least satisfied with exercise therapy/rehabilitation, irrespective of disease severity, and 31.2% of patients wished they had received more information on this topic at diagnosis. This is similar to a study on patients' needs for pharmacotherapy in Europe [15], which showed that 77.3% of patients wanted more information on exercise therapy/rehabilitation. There are long-term benefits in terms of maintaining motor function for patients with Parkinson's disease [16], and early referral to a physiotherapist is recommended [5]. However, because the benefits may be evident in the long term, more effort is required to maintain patient motivation during the early stages of physical therapy. Moreover, these findings suggest that providing patients with more information on the benefits of exercise therapy/rehabilitation and increasing the number of rehabilitation centers that can provide these services will help improve uptake and contribute to improved outcomes for patients.

Management of patients with severe Parkinson's disease symptoms or advanced disease is complex and requires a personalized approach to address motor symptoms and the nonmotor symptoms that tend to dominate in the later stages and severely impact the patient's quality of life. Although treatment options are available for some symptoms such as depression and fatigue, the available treatments for most nonmotor symptoms are inadequate [17]. In this study, almost half of the patients had severe disease (H&Y 4-5) and were likely to have multiple nonmotor symptoms; therefore, it is not unexpected that patients had lower rates of satisfaction with more severe disease and physicians had lower rates of satisfaction than patients. These findings are consistent with those from a survey of members of the Japanese Society of Neurology [18], which showed that although Japanese neurologists were mostly satisfied (92.5%) with the treatment of Parkinson's disease compared with other diseases, the proportion of those who were very satisfied (9.8%) was low. In addition, findings from a survey by Fujimoto et al. showed that patient satisfaction tended to decrease with increasing time since symptom onset, from 52.5% at <3 years to 41.0% at 3–<6 years and 37.6% at 6–>9 years [19]. In this study, in which the mean time from symptom onset was 8.3 years, patient satisfaction with pharmacotherapy was 72.7% for those with mild (H&Y 1-2) symptoms and 51.9% for those with moderate-to-severe (H&Y ≥ 3) symptoms.

Consistent with other studies [6, 11, 13], patient satisfaction with symptom (motor/nonmotor) control in this study was lower than physicians and was lower for nonmotor symptoms than motor symptoms. Several studies have shown that patients find nonmotor symptoms more difficult to alleviate [6] and place greater emphasis than physicians on nonmotor symptoms for pharmacotherapy [11, 13]. The disconnect between patients and physicians for symptom control may be because there are no established treatments for nonpsychiatric nonmotor symptoms in Parkinson's disease. Therefore, physicians may focus more on treating motor and psychiatric symptoms and overlook other nonmotor symptoms for which limited treatment options are available. In addition, patients may not realize the association between some nonmotor symptoms and Parkinson's disease and may have difficulty communicating their dissatisfaction to physicians. This is supported by our findings that nonmotor symptoms (insomnia, depression, olfactory disturbance, and fatigue) were significantly associated with lower satisfaction scores overall and that, in addition, difficulties with speech, cognitive impairment, and apathy were significantly associated with lower satisfaction scores for the consultation. Because alleviation of nonmotor symptoms is important for patient satisfaction, these findings emphasize the need for the development of effective treatments to help manage these symptoms.

It is of interest that higher rates of patient and physician satisfaction with symptom control were reported for patients who used a symptom diary than those who did not. This suggests that a symptom diary may facilitate communication between patients and physicians and be a useful tool that allows patients to monitor their symptoms outside of clinical visits. The benefits of a symptom diary are that it can provide a subjective assessment of symptoms from the patients' viewpoint and allow physicians to understand symptoms patients experience in home-based settings. As the use of both subjective and objective evaluations of patients are likely to lead to more appropriate shared decision making, it is envisaged that wearable monitoring devices that provide objective data on symptom control in patients with Parkinson's disease are likely to become important tools for personalized care in the future [20].

Improving or maintaining patient quality of life is an important management goal for chronic and slowly progressive neurodegenerative disorders such as Parkinson's disease that require long-term treatment [21]. Although it is well established that motor and nonmotor symptoms significantly affect the quality of life of patients with Parkinson's disease [22], patient satisfaction with consultation and treatment are additional contributing factors [14, 23]. As shown in this study, disconnection between patient and physician satisfaction with treatment and perceptions of management is likely to occur in diseases characterized by a broad range of symptoms that vary greatly in the way patients are affected. Physicians should be fully aware of the potential for disconnection when managing patients and should take the time needed to listen to the patient's needs, provide them with the information they require, improve links to nonspecialist support services, and involve each patient in the setting of their treatment goals. This personalized approach is likely to engage patients more in their own treatment journey, which will contribute to higher rates of satisfaction for both patients and physicians and improved patient outcomes.

Because this was a nationwide cross-sectional study conducted in real-world clinical practice, the findings from this survey may not be universally applicable to patients with PD from other countries with different cultures and healthcare systems. In addition, the mean age of patients (62.7 years) and mean age at diagnosis (56.2 years) in this study were slightly lower than those reported in studies of a Japanese Parkinson's disease registry (*n* = 23,058; age, 71.3 years (H&Y ≥ 3); disease onset, 62.7 years) [24] and from a nationwide association of patients with Parkinson's disease (*n* = 4278; age, 71.1 years for males, 70.4 years for females; disease onset, 61.5 years) [25]. These differences may be because (1) the disease registry is focused on patients with severe disease, who are more likely to be older than those with mild symptoms [24], and (2) patients in the patient association were surveyed using a paper-based questionnaire or were interviewed directly [25], which may have made it easier for older patients to participate than the current study, which required the use of the Internet. Additional sources of bias may have arisen from recall bias and the use of an Internet-based survey, where enrolled patients were Internet users and presumably had greater access to information about their disease. In addition, diagnosis and severity (using the H&Y scale) of Parkinson's disease were reported by the patients themselves or via a family member and may not accurately reflect the patient's disease state, responses, or severity of PD compared with those involved in a physician-confirmed diagnosis. We attempted to address this limitation by excluding patients who were not currently taking medication and who did not have major symptoms and by limiting responses from family members to those patients who directly confirmed the responses at the time the questionnaire was conducted. Finally, although we assessed the perceptions of both patients and physicians using the same questionnaire, the differences in patient and physician perspectives in this study might not reflect those we would have encountered had we used matched patient-physician pairs.

## 5. Conclusions

In this study, we showed that there were significant gaps between patient and physician satisfaction with treatment and perceptions of consultations at diagnosis and during routine treatment and that patient and physician satisfaction with treatment was lower for patients with moderate-to-severe symptoms than those with mild symptoms. Our analysis of the factors that contributed to patient and physician satisfaction with treatment and consultations suggests that physicians should take every opportunity to tailor treatment by listening carefully to patients' needs during consultations, encouraging the use of symptom diaries, and participating in shared decision making with their patients that includes strategies for nonmotor symptoms and nonpharmacological therapies.

## Figures and Tables

**Table 1:** Demographics of eligible respondents (patients).

Characteristic	Total, *N* = 186
Sex, male, %	54.8
Mean age, years, mean ± SD	62.7 ± 14.8
Age, %	
<50 years	19.4
50–59 years	16.1
60–69 years	27.4
70–79 years	26.3
>80 years	10.8
Years since symptom onset, mean ± SD	8.3 ± 7.8
Years since diagnosis, mean ± SD	6.5 ± 7.3
Age at PD diagnosis, years, mean ± SD	56.2 ± 16.3
Mean H&Y stage, mean ± SD	3.2 ± 1.3
H&Y stage, %^a^	
1-2	24.2
3	31.2
4-5	44.6
H&Y stage at PD diagnosis, mean ± SD^a^	2.6 ± 1.3
Currently experiencing wearing off (%)^b^	60.8^c^
Frequency of visits to a medical institution, %	
1 visit per week	14.5
1 visit every 2 weeks	12.4
1 visit per month	36.0
1 visit every 2 or 3 months	34.4
≤1 visit every 4 months	1.6
Unknown	1.1
Current treatment department, %	
Neurology	76.3
Neurosurgery	10.2
General internal medicine	4.3
Psychiatry	4.3
Other	4.8

^a^For patients, H&Y stage was self-reported; ^b^wearing off means duration of effectiveness of medication may be shorter than when the medication was effective and the symptoms of Parkinson's disease were stable; symptoms of Parkinson's disease may appear before the next administration; ^c^included patients who answered “once a day,” “twice a day,” or “≥3 times a day.” H&Y; Hoehn and Yahr; PD; Parkinson's disease; SD: standard deviation.

**Table 2:** Demographics of eligible respondents (physicians).

Characteristic	Total, *N* = 331
Sex, male, %	93.1
Age, %	
<40 years	17.2
40–49 years	29.6
50–59 years	36.0
>60 years	17.2
Clinical experience, years, mean ± SD	19.4 ± 8.7
Number of patients with PD treated (in the last 6 months), mean ± SD	52.1 ± 65.9
Certified neurologist, %	34.1
Department, %	
General internal medicine	37.5
Neurology	32.9
Neurosurgery	16.9
Psychiatry	12.7
Number of beds, %	
0	15.7
1–199	29.0
200–399	22.4
>400	32.9

PD: Parkinson's disease; SD: standard deviation.

## Data Availability

The datasets generated and/or analyzed during the current study are available from the corresponding author on reasonable request.

## Abbreviations

DBS: Deep brain stimulation
FA: Free answer
H&Y: Hoehn and Yahr
ICD: Impulse control disorders
MA: Multiple answer
N: Number
PD: Parkinson's disease
SA: Single answer
SC: Screening
SD: Standard deviation.

## Additional Points

There were significant gaps between patient and physician satisfaction with treatment and perceptions of consultations at diagnosis and during routine treatment. Patients were less satisfied with symptom control than physicians were. Patients and physicians were more satisfied with symptom control when symptom diaries were used.

## Supplementary Materials

Supplementary Materials Figure S1. Flow diagram for inclusion and exclusion criteria: A : patients; B : physicians. ^a^Physicians did not meet the following inclusion criteria: neurologists who had examined ≥25 patients with Parkinson's disease in the last 6 months; neurosurgeons who had examined ≥5 patients with Parkinson's disease in the last 6 months; and general internal physicians or psychiatrists who had examined ≥10 patients with Parkinson's disease in the last 6 months. PD = Parkinson's disease. Figure S2. Satisfaction with symptom control: A : patients (motor); B : patients (nonmotor); C : physicians (motor); and D : physicians (nonmotor). ^a^Difficulties with these functions. ICD, impulse control disorders. Table S1-1: abridged summary of the screening and main questionnaires for patients used in the study. Table S1-2: abridged summary of the screening and main questionnaires for physicians used in the study. Table S2: bivariate analyses of the associations between patient satisfaction and factors related to treatment. Table S3: bivariate analyses of the associations between physician satisfaction and factors related to treatment.
