# Supplementary material for: Treatment Satisfaction and Its Influencing Factors in Parkinson's Disease: A Web-Based Survey of Patients and Physicians in Clinical Practice in Japan
Source: Parkinsons Dis. 2022 Feb 23;2022:2732021. doi: 10.1155/2022/2732021 (PMC8890898; doi:10.1155/2022/2732021)
Supplement: Supplementary Materials — Figure S1. Flow diagram for inclusion and exclusion criteria: A : patients; B : physicians. aPhysicians did not meet the following inclusion criteria: neurologists who had examined ≥25 patients with Parkinson's disease in the last 6 months; neurosurgeons who had examined ≥5 patients with Parkinson's disease in the last 6 months; and general internal physicians or psychiatrists who had examined ≥10 patients with Parkinson's disease in the last 6 months. PD = Parkinson's disease. Figure S2. Satisfaction with symptom control: A : patients (motor); B : patients (nonmotor); C : physicians (motor); and D : physicians (nonmotor). aDifficulties with these functions. ICD, impulse control disorders. Table S1-1: abridged summary of the screening and main questionnaires for patients used in the study. Table S1-2: abridged summary of the screening and main questionnaires for physicians used in the study. Table S2: bivariate analyses of the associations between patient satisfaction and factors related to treatment. Table S3: bivariate analyses of the associations between physician satisfaction and factors related to treatment. [file 2732021.f1.zip › Supplemental Table S1_PD web survey MS_03Jan22.pdf]

TABLE S1-1: Abridged summary of the screening and main questionnaires for patients used in the study.

Screening questionnaire			
**No.**	**Category and questions**	**Answer type**	**Answer**
**Permission**			
SC1	This questionnaire contains questions about "the physical condition of you/your family member [symptoms, diseases, etc.]". Data will be used for medical activities after being statistically processed so that specific individuals cannot be identified. If you agree with our intent, please answer the questions.<br>If you decide not to answer the questions, please press the "Quit" button or close the browser to finish the questionnaire.<br>To all respondents to this questionnaire:<br>We have asked Macromill Monitors to thoroughly comply with "the obligation of confidentiality regarding the survey" in the terms for monitoring. Please do not disclose to third parties the details of this questionnaire and information you obtained in the course of answering the questions.<br>If adverse events (such as side effects) are found in specific patients in the course of proceeding with this marketing survey questionnaire and they are caused by our client's products, we are required to report the information to our client. Therefore, in the event that we need to ask your doctor about detailed information (e.g., serious adverse events), we will ask for your permission to give your name and contact information to our client at a later date.<br>If you agree to the above, please select "Agree" and proceed to the questionnaire.<br>Do you agree to the above regarding your participation in the "survey"?	SA	1. I agree<br>2. I do not agree
**Occupation**			
SC2	Do/Does you/your family member currently work in any of the following industries? Or did you/your family member previously work in any of the following industries? Please select all that apply.	MA	1. Food manufacturing/wholesale/sales<br>2. Beverage manufacturing/wholesale/sales<br>3. Tobacco manufacturing/wholesale/sales<br>4. Vehicle manufacturing/wholesale/sales<br>5. Clothes and/or shoe manufacturing/wholesale/sales<br>6. Retailer (department store, supermarket, convenience store)<br>7. Drug and/or medicine manufacturing/wholesale/sales<br>8. Cosmetics manufacturing/wholesale/sales<br>9. Chemical products manufacturing/wholesale/sales<br>10. Home appliances manufacturing/wholesale/sales<br>11. Stock company/commodity/derivatives company<br>12. Finance and insurance industry<br>13. Real estate industry<br>14. Transportation industry<br>15. IT/communication industry<br>16. Mass communications<br>17. Advertising agency<br>18. Marketing research company/Think tank<br>19. Other service industry<br>20. Healthcare/nursing/welfare industry

Screening questionnaire

No.	Category and questions	Answer type	Answer
			21. Schools/education industry<br>22. Government and public office/local government/public body<br>23. Nobody is/was engaged in the above industries
**Family members living together**			
SC3	Please let us know the family members living with you.	MA	1. Spouse<br>2. Father<br>3. Mother<br>4. Father-in-law<br>5. Mother-in-law<br>6. Sibling(s)<br>7. Grandfather<br>8. Grandmother<br>9. Child(ren)<br>10. Other relative (specifically:)<br>11. Other than a relative<br>12. No-one
**Diseases being treated**			
SC4	With regard to you/your family member living with you, please let us know all diseases for which treatment is being given by a doctor following diagnosis.	MA	

Diseases	1 Yourself	2 Spouse	3 Father	4 Mother	5 Father-in-law	6 Mother-in-law	7 Sibling(s)	8 Grand-father	9 Grand-mother	10 Child(ren)
1. Hypertension										
2. Diabetes mellitus										
3. Arteriosclerosis										
4. Cerebral infarction/stroke										
5. Insomnia										
6. Depression										
7. Dementia										
8. Parkinson's disease										
9. Low back pain/joint pain										
10. Cervical spondylosis										
11. Spinal stenosis										
12. Urinary tract infection										
13. Others										
14. There is no disease that is continuously treated										

No.	Category and questions	Answer type	Answer
**Onset of disease**			
SC5	The following question is for those who are being treated for Parkinson's disease or whose family member living with you is being treated for Parkinson's disease. Please let us know the current age of you/your family member living with you being treated for Parkinson's disease, the age when you/your family member started to be concerned about the symptoms of Parkinson's disease, the age when you/your family member started the treatment (drug), and the age when you/your family member was diagnosed with Parkinson's disease.	FA	

Screening questionnaire						
**No.**	**Category and questions**	**Answer type**	**Answer**			
	*The number of months can be approximate, but if it is not known, please leave it blank.<br>*If you have multiple family members with Parkinson's disease, please answer the question about the family member who was diagnosed most recently.		1. Yourself<br>Years / months old		2. Your family member living with you<br>Years / months old	
	1. Age when first concerned about the symptoms		( ) / ( )		( ) / ( )	
	2. Age when first visited the medical institution for examination after onset of symptoms		( ) / ( )		( ) / ( )	
	3. Age when diagnosed with Parkinson's disease at the medical institution		( ) / ( )		( ) / ( )	
	4. Age when started receiving treatment (medication) for Parkinson's disease		( ) / ( )		( ) / ( )	
**Presence or absence of symptoms**						
SC6	Please select all currently applicable symptoms of yourself/your family member living with you who is being treated for Parkinson's disease.	MA	1. Yourself		2. Your family member living with you	
	1. Tremor/shaking (unconscious shaking in hand[s], leg[s], chin, etc. at rest)		☐		☐	
	2. Slowness of movement (movement has become slow, walking speed has become slow, having difficulties turning over in bed, etc.)		☐		☐	
	3. Muscle stiffness (muscles are stiff and hard, joints do not move smoothly)		☐		☐	
	4. Postural instability (poor body balance, easily falling over or tumbling over)		☐		☐	
	5. Uncontrolled movement, writhing movement (dyskinesia)		☐		☐	
	6. Symptoms related to sleep (having difficulties falling asleep, awakening during the night)		☐		☐	
	7. Symptoms related to urination and defecation (needing to get up during the night for urination, having constipation)		☐		☐	
	8. Symptoms related to attention/memory (cannot concentrate, forgetful, etc.)		☐		☐	
	9. Symptoms related to mood and motivation (have no interest in anything, depressed feeling)		☐		☐	
	10. Symptoms related to having meals and gastrointestinal symptoms (difficulty swallowing, having nausea)		☐		☐	
	11. Symptoms related to pain (having pain associated with muscle stiffness, low back pain)		☐		☐	
	12. Symptoms related to hallucination or visual hallucination (seeing something that is not actually there)		☐		☐	
	13. Symptoms related to impulse (having impulsive behavior such as gambling, shopping, and games, having delusions or jealousy)		☐		☐	
	14. Duration of effectiveness of medication has become shorter, the effects disappear before the next administration, and the symptoms worsen		☐		☐	
	15. None applicable		☐		☐	
**Classification of symptoms (self-reported in accordance with Hoehn and Yahr)**						
SC7	With regard to you/your family member living with you being treated for Parkinson's disease, please let us know the symptoms of Parkinson's disease at diagnosis and the current symptoms.	MA	1. Yourself		2. Your family member living with you	
			At time of diagnosis	Current	At time of diagnosis	Current
	1. The symptoms (tremor or stiffness) occur only in one arm and leg.		☐	☐	☐	☐

Screening questionnaire						
**No.**	**Category and questions**	**Answer type**	**Answer**			
	2. The symptoms (tremor or stiffness) occur in both arms and legs.		☐	☐	☐	☐
	3. Difficulty maintaining posture, easily losing balance, and difficulty walking. Able to take care of oneself but activities are slightly limited.		☐	☐	☐	☐
	4. Difficulty standing up or walking, need to be partly assisted in daily life.		☐	☐	☐	☐
	5. Unable to stand up or walk independently, need to be completely assisted in daily life.		☐	☐	☐	☐
	6. Do not know		☐	☐	☐	☐
**Spending time with family member**						
SC8	Please let us know how you spend time with your family member living with you with Parkinson's disease.	SA	1. Spend time together almost every day (or providing nursing care).<br>2. Spend time together 2–3 times a week (or providing nursing care).<br>3. Spend time together once a week (or providing nursing care).<br>4. Less frequently than the above.			
**Frequency of visiting the hospital**						
SC9	With regard to you/your family member living with you being treated for Parkinson's disease, please let us know how often you/they visit the medical institution where you/they are receiving treatment and how often you/they attend for examination.	SA	Yourself	Your family member living with you		
			Frequency of hospital visits	Frequency of hospital visits	Frequency of accompanying your family member on hospital visits	
	1. Once a week		☐	☐	☐	
	2. Once every 2 weeks		☐	☐	☐	
	3. Once a month		☐	☐	☐	
	4. Once every 2–3 months		☐	☐	☐	
	5. Once every 4 months or less		☐	☐	☐	
	6. Do not know		☐	☐	☐	
	7. My family does not attend		☐	☐	☐	
**Medication**						
SC 10	With regard to you/your family member living with you being treated for Parkinson's disease, please let us know which drugs are being taken. Please let us know about all current drugs and treatments received for Parkinson's disease. Also, please let us know about drugs received for disease(s) other than Parkinson's disease.	FA				
			Yourself	Your family member living with you		
	Treatment (drug) for Parkinson's disease					
	1. Do not receive any drug		☐	☐		
	2. Take one kind of drug		☐	☐		
	3. Take 2 kinds of drug		☐	☐		
	4. Take 3 kinds of drug		☐	☐		

No.	Category and questions	Answer type	Answer	
**Screening questionnaire**				
	5. Take 4 or more kinds of drug		☐	☐
	6. Do not know		☐	☐
	Treatment (other than drug) for Parkinson's disease			
	7. Do not receive treatment other than drug		☐	☐
	8. Receive surgical therapy (DBS, etc.)		☐	☐
	9. Receive rehabilitation at the hospital or other facility		☐	☐
	10. Receive treatment other than the above		☐	☐
	11. Do not know		☐	☐
	Treatment (drug) for disease(s) other than Parkinson's disease			
	12. Do not receive any drug		☐	☐
	13. Take one kind of drug		☐	☐
	14. Take 2 kinds of drug		☐	☐
	15. Take 3 kinds of drug		☐	☐
	16. Take 4 or more kinds of drug		☐	☐
	17. Do not know		☐	☐
**Registration information**				
	Sex	SA	Male, Female	
	Age	FA	( ) years old	
**Main questionnaire**				
**Permission**				
Q1	Same as screening questions (SC1)			
**Current symptoms and condition (motor and non-motor symptoms)**				
Q2	We need to ask the following questions because you answered that you/your family member living with you (relationship) had Parkinson's disease in the "questionnaire on health (screening questionnaire)" in which you recently participated. Regarding the current condition of yourself/your family member (relationship) with Parkinson's disease, please select all the symptoms you/your family member currently have from the following.	MA		
	Motor symptoms			
	1. Tremor/shaking (unconscious shaking in hand[s], leg[s], chin, etc. at rest)			
	2. Muscle stiffness (muscles are stiff and hard, joints do not move smoothly)			
	3. Slowness of movement (movement has become slow, walking speed has slowed, having difficulties turning over in bed, etc.)			
	4. Postural instability (poor body balance, easily falling over or tumbling over)			
	5. Freezing of gait			
	6. Uncontrolled movement, writhing movement (dyskinesia)			

Screening questionnaire			
**No.**	**Category and questions**	**Answer type**	**Answer**
	7. Having difficulties writing letters, letters get smaller		
	8. Having difficulties with speech, hard to produce a sound, small voice		
	Symptoms related to sleep		
	9. Having difficulties falling asleep, awakening during the night		
	10. Drowsy during the day		
	Symptoms related to urination and defecation		
	11. Urgency for urination, needing to get up during the night for urination		
	12. Having constipation, difficulties having bowel movements		
	Symptoms related to attention/memory		
	13. Forgetful, cannot concentrate		
	Symptoms related to mood and motivation		
	14. Lack of interest, lack of motivation		
	15. Depressed, no longer enjoy what used to enjoy		
	Symptoms related to having meals and gastrointestinal symptoms		
	16. Difficulty swallowing		
	17. Upset stomach, nausea		
	Symptoms related to pain		
	18. Having pain associated with muscle stiffness, pain during off period		
	19. Having low back pain or joint pain		
	Symptoms related to hallucination or visual hallucination		
	20. Seeing something that is not actually there		
	Symptoms related to impulse		
	21. Having impulsive behavior such as gambling, shopping, and games, having jealousy or delusions, altered sexual interest		
	Other symptoms		
	22. Light-headedness when standing up		
	23. Having abnormal sense of smell or taste		
	24. Sweating excessively		
	25. Always feeling fatigued		
	26. None of the above symptoms		
**Wearing off**			
Q4	Have/Has you/your family member (relationship) with Parkinson's disease had the following experience regarding the effect of their medication from the start of the treatment (medication) to the present? Please include in your answer the frequency of the symptoms during the day.<br><br>The duration of effect of medication may be shorter than when the medication was effective and the symptoms of Parkinson's disease were stable, and symptoms of Parkinson's disease may appear before the next administration.	SA	Currently experiencing<br>1. Once a day<br>2. Twice a day<br>3. 3 or more times a day<br>Experienced in the past but not now<br>4. Once a day<br>5. Twice a day<br>6. 3 or more times a day<br>Experienced in the past but not now<br>7. Never experienced<br>8. Never treated with medication
**Visited medical institution**			

Screening questionnaire			
**No.**	**Category and questions**	**Answer type**	**Answer**
Q9	These questions are about the consultation, diagnosis, and treatment of Parkinson's disease of you/your family member (relationship).<br>Please let us know the medical institutions that you/your family member (relationship) with Parkinson's disease visited in each time period (at the first visit of the medical institution after the onset of symptoms, at the time of diagnosis of Parkinson's disease at the medical institution, at the time of starting to receive treatment for Parkinson's disease, and current).	SA	University hospital<br>1. Neurology department<br>2. Psychiatry<br>3. Internal medicine other than the above<br>4. Neurosurgery<br>5. Orthopedic surgery<br>6. Other<br>Hospital other than university hospital<br>7. Neurology department<br>8. Psychiatry<br>9. Internal medicine other than the above<br>10. Neurosurgery<br>11. Orthopedic surgery<br>12. Other<br>Clinic<br>13. Neurology department<br>14. Psychiatry<br>15. Internal medicine other than the above<br>16. Neurosurgery<br>17. Orthopedic surgery<br>18. Other<br>Other<br>19. Other
**Overall satisfaction with examination/treatment, satisfaction with consultation (time, content, and communication)**			
Q18	These questions are about the medical examination for Parkinson's disease at the medical institution. Please let us know how satisfied you/your family member with Parkinson's disease are/is with the treatment for Parkinson's disease.	SA	1. Extremely dissatisfied<br>2. Dissatisfied<br>3. Slightly dissatisfied<br>4. Slightly satisfied<br>5. Satisfied<br>6. Extremely satisfied<br>7. Not applicable
	Consultation<br>1. Consultation hours		
	2. The explanation given by the physician at the consultation (comprehensibility of explanation)		
	3. Treatment strategy/decision		
	4. Communication during the consultation (conversation, communication)		
	5. Overall consultation (considering the above 1 to 4 comprehensively)		
	Pharmacotherapy<br>6. Medication effectiveness (improvement/prevention of symptoms, time taken to be effective)		
	7. Side effects (severity, impact on daily life)		
	8. Convenience (easy to take the medication, frequency of administration)		
	9. Overall pharmacotherapy (considering the above 6 to 8 comprehensively)		

Screening questionnaire			
**No.**	**Category and questions**	**Answer type**	**Answer**
	Exercise therapy, rehabilitation		
	10. Instructions on exercise therapy/rehabilitation (comprehensibility of the explanation, frequency of instructions)		
	11. Effects of exercise therapy/rehabilitation (improvement/prevention of symptoms)		
	12. Exercise therapy/rehabilitation in general (considering the above 10 and 11 comprehensively)		
	Treatment		
	13. Overall treatment (considering the above 6 to 12 comprehensively)		
	Other		
	14. Support from healthcare workers other than physicians (nurse, pharmacist, physiotherapist, etc.)		
	15. Medical expense subsidies (for examination, medication, and rehabilitation, etc.)		
**Satisfaction with symptoms**			
Q19	Please let us know how satisfied you/your family member (relationship) with Parkinson's disease are/is with the current status of control of Parkinson's disease symptoms (one answer in each row).	SA	1. Extremely dissatisfied<br>2. Dissatisfied<br>3. Satisfied<br>4. Extremely satisfied
	Motor symptoms		
	1. Tremor/shaking (unconscious shaking in hand[s], leg[s], chin, etc. at rest)		
	2. Muscle stiffness (muscles are stiff and hard, joints do not move smoothly)		
	3. Slowness of movement (movement has become slow, walking speed has slowed, having difficulties turning over in bed, etc.)		
	4. Postural instability (poor body balance, easily falling over or tumbling over)		
	5. Freezing of gait		
	6. Uncontrolled movement, writhing movement (dyskinesia)		
	7. Having difficulties writing letters, letters get smaller		
	8. Having difficulties with speech, hard to produce a sound, small voice		
	Symptoms related to sleep		
	9. Having difficulties falling asleep, awakening during the night		
	10. Drowsy during the day		
	Symptoms related to urination and defecation		
	11. Urgency for urination, needing to get up during the night for urination		
	12. Having constipation, difficulties having bowel movements		
	Symptoms related to attention/memory		
	13. Forgetful, cannot concentrate		
	Symptoms related to mood and motivation		
	14. Lack of interest, lack of motivation		
	15. Depressed, no longer enjoy what used to enjoy		
	Symptoms related to having meals and gastrointestinal symptoms		
	16. Difficulty swallowing		
	17. Upset stomach, nausea		

Screening questionnaire					
No.	Category and questions	Answer type	Answer		
	Symptoms related to pain				
	18. Having pain associated with muscle stiffness, pain during off period				
	19. Having low back pain or joint pain				
	Symptoms related to hallucination or visual hallucination				
	20. Seeing something that is not actually there				
	Symptoms related to impulse				
	21. Having impulsive behavior such as gambling, shopping, and games, having jealousy or delusions, altered sexual interest				
	Effects of medication				
	22. Duration of effectiveness of medication may be shorter than when the medication was effective and the symptoms of PD were stable; symptoms of Parkinson's disease may appear before the next administration.				
	Other symptoms				
	23. Light-headedness when standing up				
	24. Having abnormal sense of smell or taste				
	25. Sweating excessively				
	26. Always feeling fatigued				
**Explanation topics from physicians during the consultation at PD diagnosis**					
Q20	What explanation were/was you/your family member (relationship) given by the physician at the time of diagnosis of Parkinson's disease. Please let us know all the contents of the explanation.<br>Regarding the contents of the explanation given by the physician, please let us know the details of the explanation that satisfied you/your family member.<br>Please let us know what additional explanation you wanted regardless of whether the topic was explained to you or not.	MA	1<br>The contents of the explanation (as many as you like)	2<br>The contents with a fully briefed explanation (as many as you like)	3<br>Wish to receive additional explanation (as many as you like)
	1. Causes and mechanism of the disease				
	2. Characteristic symptoms				
	3. Progression of the disease				
	4. How to deal with worsening symptoms				
	5. Effects and side effects of medication				
	6. Rehabilitation and exercise therapy (method of use, program)				
	7. Surgical treatment				
	8. What lifestyle factors patients should be aware of				
	9. Social support (care service, etc.)				
	10. Medical expense subsidies				
	11. Patients' association (how to be a member, events)				
**Contents of discussion topics during the routine examination**					
Q22	Please let us know what you/your family member (relationship) with Parkinson's disease explained to or discussed with the physician during the routine examination for Parkinson's disease. Also, please let us know the details that satisfied you/your family member about the physician's response after your explanation/discussion.	MA	1<br>Contents: explained to and discussed with the physician (select all that apply)	2<br>Contents: satisfied with physician's explanation (select all that apply)	
	1. Changes in symptoms				
	2. Appearance of new symptoms				

Screening questionnaire				
No.	Category and questions	Answer type	Answer	
	3. Impact of symptoms on daily life			
	4. Concerns and questions about symptoms			
	5. Concerns and questions about overall treatment			
	6. Setting treatment goals			
	7. Effects of medication			
	8. Side effects of medication			
	9. Forgetting to take medication			
	10. Desire to change medication			
	11. How and how often to use rehabilitation at hospital			
	12. Frequency and procedure of exercise at home			
	13. Other			
	14. Nothing discussed/nothing satisfied with			
**Contents of discussion topics about symptoms during the routine examination**				
Q23	Please let us know below everything that you/your family member explained or discussed about the symptoms of Parkinson's disease in the routine examination. Also, please let us know the details that satisfied you/your family member about the doctor's response after the consultation. Select all that apply.	MA	1<br>Contents: explained to and discussed with the physician (select all that apply)	2<br>Contents: satisfied with physician's explanation (select all that apply)
	1. Tremor/shaking (unconscious shaking in hand[s], leg[s], chin, etc. at rest)			
	2. Slowness of movement (movement and walking speed has become slow, difficulties turning over in bed, etc.)			
	3. Muscle stiffness (muscles are stiff and hard, joints do not move smoothly)			
	4. Postural instability (poor body balance, easily falling over or tumbling over)			
	5. Uncontrolled movement, writhing movement (dyskinesia)			
	6. Symptoms related to sleep (having difficulties falling asleep, awakening during the night)			
	7. Symptoms related to urination and defecation (needing to get up during the night for urination, having constipation)			
	8. Symptoms related to attention/memory (cannot concentrate, forgetful)			
	9. Symptoms related to mood and motivation (have no interest in anything, depressed feeling)			
	10. Symptoms related to having meals and gastrointestinal symptoms (difficulty swallowing, having nausea)			
	11. Symptoms related to pain (having pain associated with muscle stiffness, low back pain)			
	12. Symptoms related to hallucination or visual hallucination (seeing something that is not actually there)			
	13. Symptoms related to impulse (having impulsive behavior such as gambling, shopping, and games, having delusions or jealousy, etc.)			
	14. Duration of effectiveness of medication may be shorter than when the medication was effective and/or the symptoms of PD were stable; symptoms of Parkinson's disease may appear before the next administration.			
	15. Inconvenience in daily life (changing clothes, housework, bathing, etc.)			
	16. Falling over during daily life			
	17. None of the above is applicable			

Screening questionnaire			
**No.**	**Category and questions**	**Answer type**	**Answer**
**Symptom diary**			
Q24	Do/Does you/your family member (relationship) with Parkinson's disease use a "symptom diary (including paper diary and smartphone app)"?<br>Please let us know the status of use. (One answer each)<br>Symptom diary: diary to record the time drugs are taken or change in symptoms in a day. There are two types: paper diary and smartphone app.	SA	1. Continuously using<br>2. Sometimes using<br>3. Used in the past but not now<br>4. Know of it but never used<br>5. Don't know of the symptom diary
**Confirmation**			
Q31	You answered the questionnaire about the family member (relationship) with Parkinson's disease. Did you answer the questions after checking the answers with your family member (relationship) with Parkinson's disease?	SA	1. You answered the questions after directly hearing the answers from your family member (relationship) with Parkinson's disease<br>2. You answered the questions according to your objective impressions

DBS, deep brain stimulation; FA, free answer; MA, multiple answer; SA, single answer; SC, screening.

TABLE S1-2: Abridged summary of the screening and main questionnaires for physicians used in the study.

Screening questionnaire			
**No.**	**Category and questions**	**Answer type**	**Answer**
**Permission**			
SC1	In this questionnaire, the data will be used after being statistically processed so that individuals are not identified.<br>If adverse events (such as side effects) are found in specific patients in the course of proceeding with this market research questionnaire and they are caused by our client's products, we have to report the information to our client at their request. Thus, in the event that we need to ask you about detailed information (e.g., serious adverse events), we will ask for your permission to give your name and affiliation to our client at a later date.<br>It is prohibited to disclose the information acquired in this questionnaire to any third parties (including posting on a message board), copy the screen of the questionnaire, or copy the information by taking photos.<br>If you agree to the above, please select "Agree" and proceed to the questionnaire.	SA	1. I agree<br>2. I do not agree
**Department**			
SC2	Please let us know your department.	SA	1. General internal medicine<br>2. Neurology department<br>3. Psychiatry<br>4. Neurosurgery<br>5. Other
**Facility style**			
SC3	Please let us know the management style of your facility (one answer).	SA	1. University hospital<br>2. National or public hospital<br>3. General hospital<br>4. Clinic<br>5. Other → End
**Number of beds**			
SC4	Please advise the number of beds in your institution.	SA	1. 0<br>2. 1–19<br>3. 20–99<br>4. 100–199<br>5. 200–299<br>6. 300–399<br>7. 400–499<br>8. 500 or more
**The number of patients you have treated (total)**			
SC5	Please let us know the number of patients you have treated in the last 6 months based on the medical record (actual number). Please let us know the number of Parkinson's disease patients among them. Also, please let us know the number of patients with wearing off.	N	( ) patients
**Decision-making**			
		SA	1. Deciding the treatment strategy for Parkinson's disease by myself

Screening questionnaire			
**No.**	**Category and questions**	**Answer type**	**Answer**
SC6	Please let us know your involvement in deciding the treatment strategy for Parkinson's disease (start/change of medication, etc.).		2. Deciding the treatment strategy for Parkinson's disease after consulting my supervisory doctor, etc.<br>3. Do not decide the treatment strategy for Parkinson's disease by myself
**Society**			
SC7	Which of the following societies do you belong to?	MA	1. Japanese Society of Neurology<br>2. The Japan Neurosurgical Society<br>3. Movement Disorder Society of Japan (MDSJ)<br>4. Japanese Society of Neurological Therapeutics<br>5. Not affiliated with the above societies
**Clinical experience**			
SC9	Please let us know the number of years of your clinical experience in Parkinson's disease.	SA	( ) years
**Main questionnaire**			
**Permission**			
Q1	Same as screening questions (SC1)		
**Overall satisfaction with consultation/treatment for Parkinson's disease**			
From here onwards, we would like to ask you about the medical treatment for Parkinson's disease.			
Q15	How satisfied are you with the current treatment for your patients with Parkinson's disease?<br>Please answer according to the Hoehn and Yahr scales 1–2 or ≥3. (One answer in each item)	SA	1. Extremely dissatisfied<br>2. Dissatisfied<br>3. Slightly dissatisfied<br>4. Slightly satisfied<br>5. Satisfied<br>6. Extremely satisfied<br>7. I have no applicable patient
	Consultation		
	1. Consultation hours		
	2. The explanation given by the physician at the consultation (comprehensibility of explanation)		
	3. Treatment strategy/decision (engagement/convinced)		
	4. Communication during the consultation (conversation, communication)		
	5. Overall consultation (considering the above 1 to 4 comprehensively)		
	Pharmacotherapy		
	6. Medication effectiveness (improvement/prevention of symptoms, time taken to be effective)		
	7. Side effects (severity, impact on daily life)		
	8. Convenience (easy to take the medication, frequency of administration)		
	9. Overall pharmacotherapy (considering the above 6 to 8 comprehensively)		
	Exercise therapy, rehabilitation		
	10. Instructions on exercise therapy/rehabilitation (comprehensibility of the explanation, frequency of instructions)		
	11. Effects of exercise therapy/rehabilitation (improvement/prevention of symptoms)		

Screening questionnaire			
No.	Category and questions	Answer type	Answer
	12. Exercise therapy/rehabilitation in general (considering the above 10 and 11 comprehensively)		
	Treatment		
	13. Overall treatment (considering the above 6 to 12 comprehensively)		
Satisfaction with treatment/symptoms			
Q16	Please let us know how satisfied you are with the current status of controlling symptoms for your patients with Parkinson's disease. Please answer about the following symptoms. (One answer in each item)	SA	1. Extremely dissatisfied<br>2. Dissatisfied<br>3. Satisfied<br>4. Extremely satisfied<br>5. I have no applicable patients<br>6. I don't know
	Status of control of "movement symptoms"		
	1. Tremor at rest		
	2. Muscle rigidity (muscle stiffness, rigidity)		
	3. Slowness of movement (walking speed has slowed, having difficulties turning over in bed, etc.)		
	4. Postural instability (poor body balance, easily falling over or tumbling over)		
	5. Freezing of gait		
	6. Dyskinesia		
	7. Having difficulties writing letters (small handwriting, uneven letters)		
	8. Speech impediment (impaired voice volume, intonation, clearness)		
	Status of control of "symptoms related to sleep"		
	9. Insomnia (having difficulties falling asleep, waking up during sleep)		
	10. Drowsiness during the day		
	Status of control of "symptoms related to urination and defecation"		
	11. Urination trouble (frequent urination during the day and at night)		
	12. Constipation		
	Status of control of "symptoms related to attention/memory"		
	13. Cognitive disorder (impaired memory, attention, and information processing ability)		
	Status of control of "symptoms related to mood and motivation"		
	14. Indifference/apathy (a lack of interest)		
	15. Depressed feeling (depressed, feeling no joy)		
	Status of control of "symptoms related to having meals and gastrointestinal symptoms"		
	16. Having difficulties chewing/swallowing		
	17. Upset stomach, nausea		
	Status of control of "symptoms related to pain"		
	18. Dystonia (painful cramp in arms and legs, having pain during an off period)		
	19. Low back pain, joint pain		
	Status of control of "symptoms related to hallucination or visual hallucination"		

Screening questionnaire			
**No.**	**Category and questions**	**Answer type**	**Answer**
	20. Hallucination, visual hallucination		
	Status of control of “symptoms related to impulse”		
	21. Impulse control disorder (addicted to gambling or shopping, having jealousy or delusions, altered sexual interest)		
	Status of control of “the effects of drugs”		
	22. Wearing off phenomenon		
	Status of control of “other symptoms”		
	23. Light-headedness, wobbling		
	24. Abnormal sense of smell or taste		
	25. Sweating disorder (sweating excessively)		
	26. Fatigue (always feeling fatigued)		
**Topics of explanation at the time of diagnosis**			
Q17	What explanation do you give to patients diagnosed with Parkinson’s disease? Please let us know all the explanation given at the time of diagnosis. Please let us know the details of the explanation that satisfy the patients. Also, please let us know what you do not explain to the patients at the time of diagnosis.	MA	1. The details that you explain at the time of diagnosis<br>2. The explanation that satisfies the patients<br>3. The details that you do not explain but ask other staff to explain
	1. Causes and mechanism of the disease		
	2. Characteristic symptoms (what symptoms occur)		
	3. Progression of the disease		
	4. How to deal with worsening symptoms		
	5. Effects and side effects of medication		
	6. Rehabilitation and exercise therapy (method for use, program)		
	7. Surgical treatment		
	8. What lifestyle factors patients should be aware of		
	9. Social support (care service, etc.)		
	10. Medical expense subsidies		
	11. Patients’ association (how to be a member, events)		
	12. None of the above is applicable		
**Understanding of Parkinson’s disease (progression, future treatment, etc.)**			
Q18	How much do you think the patients understand the below details about Parkinson’s disease?	SA	1. Don’t understand at all<br>2. Don’t understand<br>3. Understand<br>4. Understand very much<br>5. Don’t know
	1. Causes and mechanism of the disease		
	2. Characteristic symptoms (what symptoms occur)		
	3. Progression of the disease		
	4. How to deal with worsening symptoms		
	5. Effects and side effects of medication		
	6. Rehabilitation and exercise therapy (method for use, program)		
	7. Surgical treatment		
	8. What lifestyle factors patients should be aware of		
	9. Social support (care service, etc.)		

Screening questionnaire				
**No.**	**Category and questions**	**Answer type**	**Answer**	
	10. Medical expense subsidies			
	11. Patients' association (how to be a member, events)			
	12. None of the above is applicable			
**What topics physicians could not check with patients during routine examination**				
Q20	Please let us know the details that you check with the patients about each symptom of Parkinson's disease below in the routine examination. Also, please let us know the details of the explanation or response that you always give after checking the patient's questions and wishes.	MA	1<br>The details that you usually check with the patient (as many as you like)	2<br>The details of the explanation or response that you always give after checking the patient's questions and wishes (as many as you like)
	1. Changes in symptoms			
	2. Appearance of new symptoms			
	3. Impact of symptoms on daily life			
	4. Concerns and questions about symptoms			
	5. Concerns and questions about overall treatment			
	6. Setting treatment goals			
	7. Effects of medication			
	8. Side effects of medication			
	9. Forgetting to take medication			
	10. Desire to change medication			
	11. How and how often to use rehabilitation at hospital			
	12. Frequency and procedure of exercise at home			
	13. Other			
	14. None of the above is applicable			
**Checking the symptoms with patients during routine examination**				
Q21	Please let us know the details you check with the patients about each symptom of Parkinson's disease below in the routine examination. Also, please let us know the details of the explanation or response that you always give after checking the patient's questions and wishes. Select all that apply.	MA	1<br>The details that you usually check with the patient (as many as you like)	2<br>The details of the explanation or response that you always give after checking the patient's questions and wishes (as many as you like)
	1. Tremor at rest			
	2. Slowness of movement (walking speed has slowed, having difficulties turning over in bed, etc.)			
	3. Muscle rigidity (muscle stiffness, rigidity)			
	4. Postural instability (poor body balance, easily falling over or tumbling over)			
	5. Dyskinesia			
	6. Insomnia (having difficulties falling asleep, waking up during sleep)			
	7. Urination trouble (frequent urination) or constipation			
	8. Cognitive disorder (impaired memory, attention, and information processing ability)			
	9. Indifference/apathy or depressed feeling			
	10. Having difficulties chewing/swallowing, or having upset stomach/nausea			
	11. Dystonia or low back pain/joint pain			

Screening questionnaire				
**No.**	**Category and questions**	**Answer type**	**Answer**	
	12. Hallucination, visual hallucination			
	13. Impulse control disorder			
	14. Wearing off phenomenon			
	15. Inconvenience in daily life (changing clothes, housework, bathing, etc.)			
	16. Falling over during daily life			
	17. None of the above is applicable			
**Use of symptom diary**				
Q22	The questions from here onwards are about the Parkinson's disease treatment support tool. Do you use “symptom diary (including paper diary and smartphone app)” for the examination of Parkinson’s disease patients? Please let us know the status of using symptom diaries.	SA	1. I instruct the patients to use it on a daily basis<br>2. I use it temporarily for the adjustment of the drug<br>3. I seldom use it<br>4. I have never used it before	
**Specialist**				
Q26	Please let us know the status of your specialist certificate from the Japanese Society of Neurology.	SA	1. I am a certified neurology specialist<br>2. I am not a specialist	
**Age group**				
Q27	Please let us know your age group.	SA	1. 20s<br>2. 30s<br>3. 40s<br>4. 50s<br>5. 60s<br>6. 70s or older	
**Sex**				
Q28	Please let us know your sex.	SA	1. Male<br>2. Female	
**Confirmation**				
Q31	Please let us know your involvement with pharmaceutical companies.	SA	1. I am affiliated with or am employed as a consultant of a pharmaceutical company<br>2. I have no employment relationship	

MA, multiple answer; N, number; SA, single answer; SC, screening.
