# Supplementary material for: Treatment Satisfaction and Its Influencing Factors in Parkinson's Disease: A Web-Based Survey of Patients and Physicians in Clinical Practice in Japan
Source: Parkinsons Dis. 2022 Feb 23;2022:2732021. doi: 10.1155/2022/2732021 (PMC8890898; doi:10.1155/2022/2732021)
Supplement: Supplementary Materials — Figure S1. Flow diagram for inclusion and exclusion criteria: A : patients; B : physicians. aPhysicians did not meet the following inclusion criteria: neurologists who had examined ≥25 patients with Parkinson's disease in the last 6 months; neurosurgeons who had examined ≥5 patients with Parkinson's disease in the last 6 months; and general internal physicians or psychiatrists who had examined ≥10 patients with Parkinson's disease in the last 6 months. PD = Parkinson's disease. Figure S2. Satisfaction with symptom control: A : patients (motor); B : patients (nonmotor); C : physicians (motor); and D : physicians (nonmotor). aDifficulties with these functions. ICD, impulse control disorders. Table S1-1: abridged summary of the screening and main questionnaires for patients used in the study. Table S1-2: abridged summary of the screening and main questionnaires for physicians used in the study. Table S2: bivariate analyses of the associations between patient satisfaction and factors related to treatment. Table S3: bivariate analyses of the associations between physician satisfaction and factors related to treatment. [file 2732021.f1.zip › Supplemental Table S2_PD web survey MS_03Jan22.pdf]

TABLE S2: Bivariate analyses of the associations between patient satisfaction and factors related to treatment.

Characteristic	Consultation		Pharmacotherapy		Exercise therapy		Medical support		Medical expense		Overall treatment	
	n	Mean ± SD	n	Mean ± SD	n	Mean ± SD	n	Mean ± SD	n	Mean ± SD	n	Mean ± SD
Age												
<60 years	66	3.76 ± 1.05	64	3.70 ± 1.12	58	2.97 ± 1.17**	62	3.45 ± 1.22	64	3.31 ± 1.30	66	3.58 ± 1.23
≥60 years	120	4.02 ± 1.28	119	3.68 ± 1.25	107	3.50 ± 1.26	106	3.66 ± 1.29	117	3.65 ± 1.32	120	3.76 ± 1.18
Sex												
Male	102	3.86 ± 1.15	100	3.64 ± 1.12	95	3.24 ± 1.22	95	3.46 ± 1.18	99	3.39 ± 1.20	102	3.56 ± 1.16
Female	83	4.01 ± 1.27	82	3.78 ± 1.27	69	3.42 ± 1.30	72	3.76 ± 1.35	81	3.72 ± 1.43	83	3.88 ± 1.22
H&Y stage												
1–2	45	4.13 ± 1.04	44	4.00 ± 0.99*	35	3.60 ± 1.24	36	3.67 ± 0.99	44	3.64 ± 1.12	45	3.98 ± 1.10
≥3	141	3.86 ± 1.25	139	3.59 ± 1.25	130	3.23 ± 1.25	132	3.56 ± 1.33	137	3.50 ± 1.37	141	3.60 ± 1.22
Job												
Yes	63	4.08 ± 1.17	62	3.77 ± 1.25	55	3.47 ± 1.12	57	3.81 ± 1.13	61	3.95 ± 1.23**	63	3.76 ± 1.19
No	123	3.85 ± 1.22	121	3.64 ± 1.18	110	3.23 ± 1.31	111	3.47 ± 1.32	120	3.32 ± 1.31	123	3.66 ± 1.21
Tremor												
Yes	116	3.81 ± 1.24	113	3.56 ± 1.19	100	3.26 ± 1.20	101	3.44 ± 1.32	113	3.42 ± 1.36	116	3.63 ± 1.19
No	70	4.11 ± 1.12	70	3.90 ± 1.21	65	3.38 ± 1.33	67	3.81 ± 1.14	68	3.71 ± 1.22	70	3.80 ± 1.21
Rigidity												
Yes	107	3.92 ± 1.32	105	3.52 ± 1.29*	99	3.31 ± 1.27	100	3.50 ± 1.36	106	3.42 ± 1.39	107	3.70 ± 1.29
No	79	3.94 ± 1.04	78	3.91 ± 1.05	66	3.30 ± 1.23	68	3.71 ± 1.11	75	3.68 ± 1.20	79	3.68 ± 1.07
Bradykinesia												
Yes	127	3.90 ± 1.24	126	3.60 ± 1.21	114	3.30 ± 1.23	116	3.58 ± 1.30	125	3.53 ± 1.35	127	3.69 ± 1.16
No	59	3.98 ± 1.14	57	3.88 ± 1.18	51	3.33 ± 1.31	52	3.60 ± 1.19	56	3.54 ± 1.25	59	3.71 ± 1.29
Postural instability												
Yes	117	3.81 ± 1.26	116	3.56 ± 1.25	106	3.20 ± 1.29	110	3.48 ± 1.33	114	3.38 ± 1.39*	117	3.57 ± 1.18
No	69	4.12 ± 1.09	67	3.91 ± 1.08	59	3.51 ± 1.17	58	3.78 ± 1.11	67	3.79 ± 1.14	69	3.90 ± 1.21
Freezing of gait												
Yes	76	3.74 ± 1.27	76	3.42 ± 1.20*	71	3.17 ± 1.31	71	3.49 ± 1.41	75	3.51 ± 1.40	76	3.50 ± 1.22
No	110	4.05 ± 1.15	107	3.88 ± 1.17	94	3.41 ± 1.20	97	3.65 ± 1.15	106	3.55 ± 1.26	110	3.83 ± 1.17
Dyskinesia												
Yes	52	3.69 ± 1.35	51	3.24 ± 1.11**	50	3.12 ± 1.24	51	3.35 ± 1.32	51	3.27 ± 1.34	52	3.48 ± 1.21
No	134	4.01 ± 1.14	132	3.86 ± 1.20	115	3.39 ± 1.25	117	3.68 ± 1.23	130	3.63 ± 1.29	134	3.78 ± 1.19
Difficulty with handwriting												
Yes	82	3.80 ± 1.28	80	3.54 ± 1.26	73	3.21 ± 1.20	75	3.47 ± 1.34	80	3.49 ± 1.33	82	3.62 ± 1.21
No	104	4.02 ± 1.14	103	3.81 ± 1.15	92	3.39 ± 1.29	93	3.68 ± 1.20	101	3.56 ± 1.31	104	3.75 ± 1.19
Difficulty with speech												
Yes	83	3.69 ± 1.34*	82	3.38 ± 1.29**	77	3.22 ± 1.24	78	3.40 ± 1.30	82	3.43 ± 1.31	83	3.51 ± 1.19
No	103	4.12 ± 1.05	101	3.94 ± 1.07	88	3.39 ± 1.26	90	3.74 ± 1.21	99	3.62 ± 1.31	103	3.84 ± 1.19
Insomnia												
Yes	99	3.70 ± 1.31**	96	3.49 ± 1.33*	88	3.09 ± 1.31*	87	3.22 ± 1.32**	96	3.17 ± 1.34**	99	3.45 ± 1.30**
No	87	4.18 ± 1.02	87	3.91 ± 1.01	77	3.56 ± 1.14	81	3.98 ± 1.07	85	3.94 ± 1.17	87	3.97 ± 1.02

Characteristic	Consultation		Pharmacotherapy		Exercise therapy		Medical support		Medical expense		Overall treatment	
	n	Mean ± SD	n	Mean ± SD	n	Mean ± SD	n	Mean ± SD	n	Mean ± SD	n	Mean ± SD
Daytime sleepiness												
Yes	89	3.82 ± 1.29	87	3.37 ± 1.28**	80	3.16 ± 1.24	81	3.43 ± 1.31	87	3.44 ± 1.41	89	3.58 ± 1.26
No	97	4.02 ± 1.12	96	3.98 ± 1.06	85	3.45 ± 1.26	87	3.72 ± 1.21	94	3.62 ± 1.22	97	3.79 ± 1.14
Urination												
Yes	92	3.79 ± 1.34	90	3.39 ± 1.31**	77	3.19 ± 1.27	77	3.32 ± 1.36*	89	3.38 ± 1.40	92	3.51 ± 1.28*
No	94	4.05 ± 1.05	93	3.98 ± 1.01	88	3.41 ± 1.24	91	3.80 ± 1.14	92	3.67 ± 1.21	94	3.87 ± 1.09
Constipation												
Yes	90	3.82 ± 1.24	88	3.45 ± 1.25*	81	3.19 ± 1.31	81	3.49 ± 1.24	88	3.45 ± 1.33	90	3.63 ± 1.20
No	96	4.02 ± 1.17	95	3.91 ± 1.12	84	3.43 ± 1.19	87	3.67 ± 1.29	93	3.60 ± 1.30	96	3.75 ± 1.20
Cognitive impairment												
Yes	75	3.63 ± 1.33**	74	3.26 ± 1.30**	71	3.24 ± 1.38	70	3.46 ± 1.33	75	3.29 ± 1.43*	75	3.49 ± 1.35
No	111	4.13 ± 1.07	109	3.98 ± 1.04	94	3.36 ± 1.15	98	3.67 ± 1.22	106	3.70 ± 1.20	111	3.83 ± 1.07
Apathy												
Yes	56	3.63 ± 1.54*	54	3.13 ± 1.29**	52	3.06 ± 1.33	51	3.22 ± 1.40*	55	3.45 ± 1.44	56	3.45 ± 1.41
No	130	4.05 ± 1.01	129	3.92 ± 1.09	113	3.42 ± 1.20	117	3.74 ± 1.17	126	3.56 ± 1.26	130	3.80 ± 1.08
Depression												
Yes	79	3.63 ± 1.34**	77	3.30 ± 1.27**	74	3.04 ± 1.32*	72	3.33 ± 1.34*	78	3.24 ± 1.37*	79	3.43 ± 1.31**
No	107	4.14 ± 1.05	106	3.97 ± 1.07	91	3.53 ± 1.16	96	3.77 ± 1.17	103	3.75 ± 1.23	107	3.89 ± 1.08
Difficulty swallowing												
Yes	53	3.81 ± 1.49	51	3.35 ± 1.43*	49	3.41 ± 1.31	50	3.34 ± 1.44	52	3.17 ± 1.52*	53	3.66 ± 1.39
No	133	3.97 ± 1.07	132	3.82 ± 1.08	116	3.27 ± 1.23	118	3.69 ± 1.17	129	3.67 ± 1.20	133	3.71 ± 1.12
Stomach upset												
Yes	44	3.39 ± 1.45**	42	3.07 ± 1.35**	40	3.00 ± 1.38	40	3.03 ± 1.33**	43	2.95 ± 1.40**	44	3.20 ± 1.37**
No	142	4.09 ± 1.07	141	3.87 ± 1.09	125	3.41 ± 1.20	128	3.76 ± 1.20	138	3.71 ± 1.24	142	3.85 ± 1.10
Dystonia												
Yes	68	3.74 ± 1.27	66	3.41 ± 1.18*	63	3.14 ± 1.18	64	3.45 ± 1.31	67	3.33 ± 1.34	68	3.51 ± 1.25
No	118	4.03 ± 1.16	117	3.85 ± 1.19	102	3.41 ± 1.29	104	3.66 ± 1.24	114	3.65 ± 1.29	118	3.80 ± 1.16
Back pain and joint pain												
Yes	79	3.86 ± 1.37	76	3.61 ± 1.39	65	3.12 ± 1.32	68	3.44 ± 1.39	75	3.35 ± 1.33	79	3.70 ± 1.36
No	107	3.97 ± 1.08	107	3.75 ± 1.06	100	3.43 ± 1.20	100	3.68 ± 1.17	106	3.66 ± 1.29	107	3.69 ± 1.07
Hallucination												
Yes	40	3.73 ± 1.52	40	3.23 ± 1.40**	39	2.97 ± 1.50	38	3.24 ± 1.50	40	3.35 ± 1.64	40	3.38 ± 1.39
No	146	3.98 ± 1.10	143	3.82 ± 1.11	126	3.41 ± 1.15	130	3.68 ± 1.17	141	3.58 ± 1.21	146	3.78 ± 1.13
ICD												
Yes	35	3.66 ± 1.43	33	3.27 ± 1.46*	32	2.91 ± 1.33*	32	3.00 ± 1.32**	34	3.12 ± 1.51*	35	3.40 ± 1.40
No	151	3.99 ± 1.14	150	3.78 ± 1.12	133	3.41 ± 1.22	136	3.72 ± 1.22	147	3.63 ± 1.25	151	3.76 ± 1.14
Dizziness												
Yes	61	3.74 ± 1.39	60	3.57 ± 1.42	55	3.07 ± 1.36	59	3.56 ± 1.42	59	3.31 ± 1.52	61	3.51 ± 1.37
No	125	4.02 ± 1.10	123	3.75 ± 1.08	110	3.43 ± 1.18	109	3.60 ± 1.18	122	3.64 ± 1.19	125	3.78 ± 1.10
Olfactory disturbances												
Yes	30	3.13 ± 1.50**	30	2.87 ± 1.33**	29	3.14 ± 1.36	29	2.79 ± 1.42**	30	2.97 ± 1.59**	30	3.07 ± 1.31**

Characteristic	Consultation		Pharmacotherapy		Exercise therapy		Medical support		Medical expense		Overall treatment	
	n	Mean ± SD	n	Mean ± SD	n	Mean ± SD	n	Mean ± SD	n	Mean ± SD	n	Mean ± SD
No	156	4.08 ± 1.08	153	3.85 ± 1.11	136	3.35 ± 1.23	139	3.75 ± 1.17	151	3.64 ± 1.23	156	3.81 ± 1.14
Sweating												
Yes	38	3.74 ± 1.35	36	3.31 ± 1.14*	33	3.12 ± 1.34	34	3.35 ± 1.39	36	3.00 ± 1.39**	38	3.58 ± 1.20
No	148	3.97 ± 1.17	147	3.78 ± 1.20	132	3.36 ± 1.23	134	3.64 ± 1.23	145	3.66 ± 1.27	148	3.72 ± 1.20
Fatigue												
Yes	77	3.55 ± 1.35**	75	3.19 ± 1.23**	71	2.96 ± 1.37**	70	3.23 ± 1.31**	76	3.22 ± 1.33**	77	3.34 ± 1.31**
No	109	4.19 ± 1.01	108	4.04 ± 1.06	94	3.57 ± 1.09	98	3.84 ± 1.17	105	3.75 ± 1.26	109	3.94 ± 1.04

*p<0.05, **p<0.01, significant difference between categories using Student *t* test.

H&Y, Hoehn and Yahr; ICD, impulse control disorders; SD, standard deviation.
