# Supplementary material for: Treatment Satisfaction and Its Influencing Factors in Parkinson's Disease: A Web-Based Survey of Patients and Physicians in Clinical Practice in Japan
Source: Parkinsons Dis. 2022 Feb 23;2022:2732021. doi: 10.1155/2022/2732021 (PMC8890898; doi:10.1155/2022/2732021)
Supplement: Supplementary Materials — Figure S1. Flow diagram for inclusion and exclusion criteria: A : patients; B : physicians. aPhysicians did not meet the following inclusion criteria: neurologists who had examined ≥25 patients with Parkinson's disease in the last 6 months; neurosurgeons who had examined ≥5 patients with Parkinson's disease in the last 6 months; and general internal physicians or psychiatrists who had examined ≥10 patients with Parkinson's disease in the last 6 months. PD = Parkinson's disease. Figure S2. Satisfaction with symptom control: A : patients (motor); B : patients (nonmotor); C : physicians (motor); and D : physicians (nonmotor). aDifficulties with these functions. ICD, impulse control disorders. Table S1-1: abridged summary of the screening and main questionnaires for patients used in the study. Table S1-2: abridged summary of the screening and main questionnaires for physicians used in the study. Table S2: bivariate analyses of the associations between patient satisfaction and factors related to treatment. Table S3: bivariate analyses of the associations between physician satisfaction and factors related to treatment. [file 2732021.f1.zip › Supplemental Table S3_PD web survey MS_03Jan22 (1).pdf]

TABLE S3: Bivariate analyses of the associations between physician satisfaction and factors related to treatment.

Characteristic	Consultation		Pharmacotherapy		Exercise therapy		Overall treatment	
	n	Mean ± SD	n	Mean ± SD	n	Mean ± SD	n	Mean ± SD
**H&Y stage (1–2)**								
Age								
≥50 years	176	3.95 ± 0.83	176	3.85 ± 0.81	172	3.74 ± 0.90*	176	3.86 ± 0.78
<50 years	155	3.99 ± 0.91	155	3.99 ± 0.91	153	3.95 ± 0.90	155	3.97 ± 0.86
Clinical experience								
≥20 years	186	3.98 ± 0.86	186	3.96 ± 0.86	183	3.78 ± 0.89	186	3.92 ± 0.78
<20 years	145	3.95 ± 0.92	145	3.86 ± 0.87	142	3.92 ± 0.92	145	3.90 ± 0.87
Department								
Neurology[a]	165	4.03 ± 0.82	165	3.99 ± 0.86	164	3.89 ± 0.93	165	4.03 ± 0.81
General internal medicine[b]	166	3.91 ± 0.95	166	3.84 ± 0.86	161	3.78 ± 0.87	166	3.80 ± 0.81
Neurology specialist								
Yes	113	4.23 ± 0.82**	113	4.20 ± 0.86**	111	4.06 ± 0.86**	113	4.18 ± 0.79**
No	218	3.83 ± 0.89	218	3.77 ± 0.83	214	3.72 ± 0.91	218	3.78 ± 0.80
Hospital								
Hospital	264	3.99 ± 0.90	264	3.95 ± 0.87	261	3.87 ± 0.91	264	3.93 ± 0.84
Clinic	67	3.90 ± 0.82	67	3.79 ± 0.81	64	3.69 ± 0.87	67	3.84 ± 0.75
PD patients (last 6 months)								
≥50 patients	129	4.25 ± 0.93**	129	4.16 ± 0.91**	128	4.07 ± 0.87**	129	4.13 ± 0.80**
<50 patients	202	3.79 ± 0.81	202	3.76 ± 0.80	197	3.69 ± 0.89	202	3.77 ± 0.80
Medical association[c]								
Member	232	4.12 ± 0.86**	232	4.02 ± 0.89**	230	3.95 ± 0.90**	232	4.05 ± 0.82**
Non-member	99	3.62 ± 0.85	99	3.67 ± 0.74	95	3.56 ± 0.86	99	3.60 ± 0.74
**H&Y stage (≥3)**								
Age								
≥50 years	176	3.55 ± 0.90	176	3.35 ± 0.92**	174	3.26 ± 0.95**	176	3.36 ± 0.95
<50 years	155	3.57 ± 1.07	155	3.63 ± 0.99	152	3.61 ± 1.00	155	3.52 ± 0.99
Clinical experience								
≥20 years	186	3.60 ± 0.94	186	3.45 ± 0.94	185	3.35 ± 0.96	186	3.44 ± 0.98
<20 years	145	3.51 ± 1.03	145	3.52 ± 0.99	141	3.52 ± 1.03	145	3.43 ± 0.96
Department								

Neurology[a]	165	3.64 ± 0.90	165	3.50 ± 0.95	164	3.46 ± 0.96	165	3.52 ± 0.97
General internal medicine[b]	166	3.48 ± 1.05	166	3.46 ± 0.97	162	3.38 ± 1.02	166	3.35 ± 0.97
Neurology specialist								
Yes	113	3.87 ± 0.90**	113	3.77 ± 0.89**	113	3.72 ± 0.97**	113	3.75 ± 0.93**
No	218	3.40 ± 0.98	218	3.33 ± 0.97	213	3.27 ± 0.97	218	3.27 ± 0.95
Hospital								
Hospital	264	3.61 ± 1.01	264	3.54 ± 0.97*	261	3.51 ± 0.99**	264	3.50 ± 0.99**
Clinic	67	3.37 ± 0.85	67	3.24 ± 0.87	65	3.09 ± 0.93	67	3.16 ± 0.86
PD patients (last 6 months)								
≥50 patients	129	3.82 ± 0.96**	129	3.79 ± 0.93**	129	3.71 ±1.03**	129	3.74 ± 0.99**
<50 patients	202	3.40 ± 0.96	202	3.28 ± 0.93	197	3.23 ± 0.92	202	3.23 ± 0.90
Medical association[c]								
Member	232	3.68 ± 0.95**	232	3.56 ± 0.95*	231	3.56 ± 0.97**	232	3.56 ± 0.97**
Non-member	99	3.28 ± 1.01	99	3.28 ± 0.96	95	3.08 ± 0.96	99	3.13 ± 0.91

*p<0.05, **p<0.01, significant difference between categories using student t-test.
[a] Neurology and neurosurgery.
[b] Internal medicine and psychiatry.
[c] A member of the Japanese Society of Neurology, the Japan Neurosurgical Society, Movement Disorder Society of Japan, or Japanese Society of Neurological Therapeutics.
H&Y = Hoehn and Yahr, PD = Parkinson’s disease, SD = standard deviation.
